# First record of the genus *Tetracona* Meyrick (Lepidoptera, Crambidae) from China, with description of a new species

**DOI:** 10.3897/zookeys.941.49559

**Published:** 2020-06-16

**Authors:** Lu-Lan Jie, Jing-Bo Yang, Wei-Chun Li

**Affiliations:** 1 College of Agronomy, Jiangxi Agricultural University, Nanchang, 330045, China Jiangxi Agricultural University Nanchang China

**Keywords:** China, Pyraloidea, Spilomelinae, snout moths, *
Tetracona
*, taxonomy

## Abstract

The genus *Tetracona* has two species with an Australian distribution. The present study aims to record the genus from China for the first time and to add a third species, *T. multispina* Jie & Li, **sp. nov.** to the genus. The new species can be distinguished from the congeners by the antemedial line connecting the postmedial line near the dorsum in the hindwing, and the phallus with a cluster of spine-like cornuti in the male genitalia. Images of the habitus, tympanal organs and male genitalia are provided for the new species.

## Introduction

The genus *Tetracona* was erected by Meyrick in 1884 with *Aediodes amathealis* Walker, 1859 as type species (Meyrick 1884). Then it was defined as a junior synonym of *Agrotera* Schrank, 1802 based on the external characters by Hampson (1899). However, the dissected structures of the males provide more effective characters to separate the two genera. Thus, Chen et al. (2017) removed it from synonymy with *Agrotera* and reinstated it as a valid genus using the male genital characters.

Before this study, the genus contained two species with an Australian distribution (Walker 1859; Meyrick 1884; Warren 1896; Hampson 1899; Chen et al. 2017). In the present paper, we record the genus in the Chinese fauna for the first time and add a new species.

## Materials and methods

The specimens were collected at night with a mercury-vapor lamp. The specimens were prepared referring to the method shown in Landry and Landry (1994). The morphological terminology follows Maes (1995). The images of the habitus and genitalia were taken using a digital camera attached to a Zeiss SteREO Discovery V12 microscope and an Optec BK-DM320 microscope, respectively. All the studied specimens are deposited in the Museum, Jiangxi Agricultural University, Nanchang, China (**JXAUM**).

## Taxonomy

### Tetracona

Taxon classificationAnimaliaLepidopteraCrambidae

Meyrick, 1884

99306ADF-98B2-5BB4-89E1-4DF07501F446

Tetracona Meyrick, 1884: 307; Chen et al. 2017: 215. Type species: Aediodes amathealis Walker, 1859, by monotypy.

#### Differential diagnosis.

The species of *Tetracona* Meyrick, 1884 are similar to the members of *Agrotera* Schrank, 1802 in their external characters. However, they can be easily distinguished from the latter by using the male genitalia: The uncus of *Tetracona* is lobe-shaped, laterally covered with dense setae; the valvae are basally equipped with a bundle bristles near the middle, and are elliptical with blunt rounded apices. In *Agrotera*, the uncus is short to elongate and conical, set with few setae; the valvae have a large, hook-like process near the base, and are elliptical with narrow and pointed apices (Chen et al. 2017).

#### Distribution.

Australia, China.

#### Remarks.

This genus is recorded from China for the first time herein.

### Key to species of *Tetracona* based on wing pattern and male genitalia

**Table:** 

1	Forewings with basal half yellow and decorated with a brown dot near basal middle (Chen et al. 2017: fig. 16)	***T. pictalis***
–	Forewings with approximately basal third yellowish white and sprinkled with orange scales, without brown dot	**2**
2	Forewings with a crescent-shaped distal discoidal stigma, postmedial line dentated outwards at approximately dorsal fourth; apical third of valva subtriangular, apex much narrower than valval base, costa concave near middle (Chen et al. 2017: figs 15, 19)	***T. amathealis***
–	Forewings with an ovate distal discoidal stigma, postmedial line distinctively incurved at approximately dorsal third; apical third of valva subrectangular, apex much wider than valval base, costa straight (Figs 1, 4)	***T. multispina* sp. nov.**

### Tetracona multispina

Taxon classificationAnimaliaLepidopteraCrambidae

Jie & Li sp. nov.

7B48B5DB-B4D2-589D-A04A-8883658108E4

http://zoobank.org/A6316933-1CD5-4AF0-9633-208BFE743B8E

Figures 1–6

#### Type material.

***Holotype*** ♂: China: Huangzihao, Fuliang (29°15'N, 117°09'E), Jiangxi Province, 220 m, 26.v.2012, Wei-Chun Li leg., genitalia slide no. JL19103 (JXAUM). ***Paratypes***: China: 1 ♂, same data as holotype, genitalia slide no. JL16099; 1 ♂, Tongboshan (28°15'N, 117°07'E), Jiangxi Province, 900 m, 30.viii.2012, Wei-Chun Li leg., genitalia slide no. JL16098; 1 ♂, Wuyuan, Shangbao (29°09'N, 117°30.6'E), Jiangxi Province, 23–28.vi.1989, Guang-Pu Shen leg.; 1 ♂, Dabali, Xunwu (29°09'N, 117°30.6'E), Jiangxi Province, 550 m, 22.vii.2007, Yu-Jian Lin leg., genitalia slide no. JL16094; 1 ♂, Doushui (29°09'N, 117°30.6'E), Shangyou, Jiangxi Province, 150 m, 20.x.1991, Yu-Jian Lin leg., genitalia slide no. JL19104; 1 ♂, Shangyou Arboretum (29°09'N, 117°30.6'E), Jiangxi Province, 230 m, 22.x.1991, Yu-Jian Lin leg., genitalia slide no. JL19104 (JXAUM).

#### Differential diagnosis.

This new species can be distinguished from its congeners by the unique characters in the hindwing and male genitalia: its antemedial line connects with the postmedial line near the dorsum and the phallus with a cluster of spine-like cornuti.

#### Description.

**Figures 1–6.**
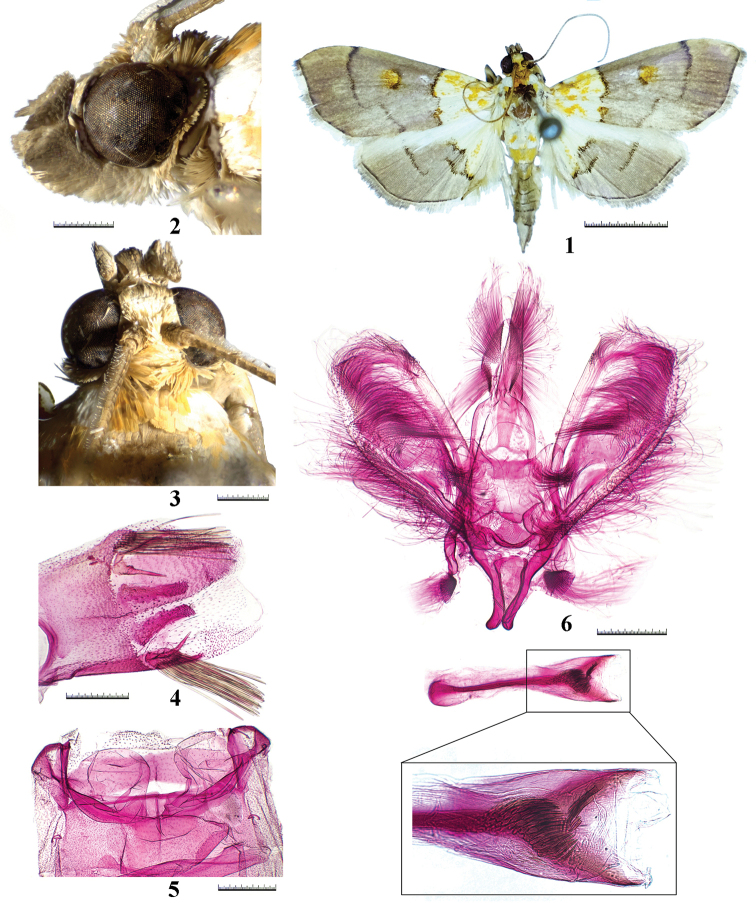
*Tetracona multispina* sp. nov. **1** adult in dorsal view, holotype **2** head in lateral view, holotype **3** head in dorsal view, holotype **4** ninth segment of abdomen, paratype **5** tympanal organs in ventral view, paratype **6** male genitalia in ventral view (phallus removed), paratype. Scale bars: 5 mm (**1**), 0.5 mm (**2–6**)

***Adult male*** (Figs 1–4): Forewing length 10.0–11.0 mm. Frons rounded, pale yellow. Vertex ocherous. Labial palpi upcurved, first segment grey, the remaining brown; second segment ending with truncate tip, third segment with triangular scale tuft. Maxillary palpi upright, ocherous. Thorax yellowish white sprinkled with orange scales. Forewing subtriangular, basal third yellowish white, suffused with irregular orange scales, remaining pale brown; antemedial line blackish brown, dentated inwards near middle; distal discoidal stigma ovate, blackish brown tinged with orange; postmedial line blackish brown, distinctively incurved at approximately dorsal third; terminal margin blackish brown; cilia pale brown mixed with pale yellow. Hindwing basal third yellowish white, suffused with irregular orange scales, remaining pale brown; antemedial line blackish brown, incurve at middle; postmedial line blackish brown, nearly S-shaped, connecting antemedial line near dorsum; terminal margin blackish brown; cilia pale brown. Abdomen with two white basal segments, second segment with two orange lateral stripes; third segment orange, remainder pale brown mixed with pale yellow except for white distal segment; ninth segment with two well-developed spines and two tufts of culcita. ***Tympanal organs*** (Fig. 5): Bulla tympani convex on inner margin, more or less concave posteriorly. Saccus tympani extending to about anterior one-fourth of tergite two. Venula secunda absent. ***Male genitalia*** (Fig. 6): Uncus lobe-shaped, covered with dense setae; distal half narrowed towards blunted tip. Valva basally narrow, broadened towards distal third, then gently narrowed towards round apex. Sacculus weakly sclerotized, thin and long. Saccus basally broad, tapering towards two blunt tips. Juxta nearly fan-shaped. Phallus straight, nearly as long as valva; cornuti composed of multiple spines of various sizes.

**Female.** Unknown.

#### Distribution.

China (Jiangxi).

#### Etymology.

The specific name is derived from the Latin prefix *multi*- = multiple, and the Latin *spina* = spine, referring to the male genitalia with multiple spine-like cornuti.

## Supplementary Material

XML Treatment for Tetracona

XML Treatment for Tetracona multispina
